# A Hydro-Mechanical Theory of Sensitive Dentin

**Published:** 1900-02-15

**Authors:** G. S. Junkerman

**Affiliations:** Cincinnati


### THE DENTAL REGISTER.

Vol. LIV. FEBRUARY 15, 1900. No. 2.

### A HYDRO-MECHANICAL THEORY OF SENSITIVE DENTIN.

BY G. S. JUNKERMAN D. D. S., CINCINNATI.

Read before the Ohio State Dental Society, December, 1899.

(*Continued from January No., page* 50.)

results and in reality becomes a curative treatment of that condition, closing up the osmotic surface by compression. Nerve fibrillæ will not account for all the phenomena of sensitive dentin, nor are their existence consistent with the comprehension of a system of nutrition and circulation in the dentin and enamel. We do not comprehend this circulation as one of a general nature, causing the waste and repair in the tissues of the teeth in general, but rather of a special nature, but nevertheless a circulation; just such a circulation as occurs in the terminal tissues in every part of the human body, a circulation by osmosis where the fluids are converted into the special tissues for which they are intended. That during the life of the pulp the dentin and enamel are supplied with nutritive fluid can not be denied, conversely after the death of the pulp these tissues manifest symptoms of inanition characterized by lack of translucency of enamel and increased chalkiness of the dentin. The same condition exists in old dentin and enamel where the dental tubuli have filled with lime salts and where the circulation has become wholly or partially strangulated, the pulp still maintaining its vitality. If we admit of a circulation in the enamel and dentin we have sufficient data to admit of a demonstration, without nerve fibrillæ, of sensitive dentin on the principle of hydro-statics and hydro-dynamics. Upon this theory it is of interest to examine the phenomena of sensitive dentin.

The greatest sensibility is found at the periphery or the line of junction between the enamel and dentin, because here we find the greatest number of terminals of tubules. Force produced at this point becomes magnified because it is transmitted to a greater number of tubules, and the pressure or motion of the fluid is in proportion to the number of tubes impressed. The periphery is at the greatest distance from the pulp, and the pressure made here must be distributed through a greater number of particles of fluid and consequently magnified proportionately when it reaches the pulp. The force increases in direct proportion to the length of the tubes. Sensitive dentin is found most frequently and to the greatest extent in well-organized teeth. In these cases we find a rank growth of the dental tubuli. There are a great number of tubules and there is also a greater tendency to anastomotic condition, the formation of a net work, radiating toward the periphery. This condition increases the length of the tubule, and consequently produces greater disturbance when force is applied. Conversely a poorly-organized tooth is less prone to sensitive dentin. The tubules are straighter and consequently shorter. There are also not so many of them and those that are may be broken by defective tracts that so frequently appear in poorly-organized dentin. It does not necessarily follow that all well-organized teeth have sensitive dentin, nor that all weaklyorganized teeth have lack of sensibility. The dental tubuli may have been infiltrated by calcareous matter thereby destroying the fluid continuity, and in such cases well-organized teeth may be operated upon without pain.

Poorly-organized dentin may be extremely sensitive, not alone on account of the delicate state of general health increasing the susceptibility, but because the softened tissues permit more easily a slight amount of force to be distributed to the greater number of tubuli. As an example of this condition, cervical cavities containing a layer of softened dentin are frequently sensitive only until this layer is removed. The application of the instrument to any part of the layer distributes the force through the entire surface which this layer covers. The therapy of sensitive dentin indicates the hydro theory to be correct. Heat and those other agents that are absorbers of moisture have proven the most beneficial ones. Anodynes not possessing these qualities have been failures. Cocain and cataphoresis should have anesthetized the nerve fibrillæ, but water and cataphoresis have been just as effective. The result in both cases was an osmosis, but the failure of any wonderful end, except to eventually destroy the real seat of sensitive dentin, the pulp. We have the remedies for sensitive dentin in anhydrous sulphuric acid, but other drugs in milder cases answer quite as well, but in the case of the stronger medicine we have not the power to limit the effect. Osmosis once established in the strata of dentin is not easily accessible to interruption. We look upon the fluid in the dental tubuli as not only a circulatory fluid for nutrition, as the blood for other tissues, but as the carrier of impressions accomplished by the nerves in other tissues. The equilibrium of these fluids is maintained by capillarity, disturbed by chemical reactionary changes and also by mechanical force; thus all this force is produced by the pressure or motion of fluid in tubes, and owes its distribution to the laws of hydro-statics and hydro-dynamics.

DISCUSSION.

Dr. C. T. Whinery, Toledo: In opening the discussion on this paper I will begin by quoting the doctor. "No theory, however, is tenable that does not satisfy all the phenomena connected with it." The theory, as elaborated, does not explain all the phenomena connected with it. For instance, the doctor says "the tract of the dentin being an osmotic membrane." If this is true the doctor's theory is exploded, as he states in his last sentence that the pain is due wholly to hydro-dynamical causes. If it is osmotic (the interchange of liquids of a different density) then certainly and positively the chemical, acid, salt, etc., can stimulate the nerve endings by the commonest of all nerve stimulant, a metabolic or chemical action.

Again, the doctor says: "Impressions must be conveyed to the pulp, which is the seat of all sensation in the teeth, by means of hydro-mechanics." I have a patient who has four Logan crowns who complains of a sensation about them when he takes hot liquids, acids, etc. The theory of the doctor that sensation is due to the increased pressure does not explain this cause, while the theory that sensation in teeth may be caused by either mechanical or chemical changes, setting up metabolism which is the origin of all sensation in nerves, explains this case perfectly.

Again, quoting the doctor: "If you put a bundle of these tubes together you have an osmotic membrane upon which impressions outward or inward may be made by any substance that will induce osmosis." I should answer if it is osmosis then the theory of chemical stimulation is not only tenable but probable. If it is merely hydro-mechanics, the term osmosis is incorrectly used.

The following explains the doctor's statement that continued use of acid is not painful. No, it is not, because we have stimulated and continued to present the stimulus which began it, and thus the nerve does not present further evidence of irritation (as electrical stimulation for extraction and sciatica) until we present a stimulus whose katabolic or nerve stimulating action differs from that in use.

Again, the doctor speaks of a steel instrument placed on the "on-edge" surface of a tooth causing no pain, and exerting a curative influence by preventing osmosis. If the argument were consistent that pain is caused by pressure or traction on nerve endings, the pressure would increase the pain. My theory is this, that if the pulp is healthy, an ordinarily cool instrument would be within the normal temperature range of the teeth and no chemical action taking place, there would be no stimulation and no pain.

The doctor speaks of a circulation in the enamel. We know that many support the same view, for the microscope reveals markings between the individual elements and prisms. But it is proven that the enamel contains only 2 or 3 percent of organic matter, and this, according to Tomes, is found to be mostly water, and if any activity occurs in this structure it must be of low grade. Let us concede, for further argument, that there is a circulation in the enamel. Why is not the enamel sensitive as well as the dentin? The enamel circulation must necessarily be a part of and a continuation of the dentinal system, and consequently irritation would be carried to the pulp almost as readily as if the cause occurred in the dentin.

Further, the doctor says: "Force produced at the periphery of the dentin is magnified, because it is transmitted to a greater number of tubules." Force can not be magnified; it really is lessened by transmission to a greater number of tubules and greater the loss occurs.

Is sensitive dentin found most frequently and to the greater degree in well-organized teeth? Does not nature tend to overcome poorly-organized teeth by following the established laws of bone pathology in other parts of the body by opening up new channels or lacunæ as receptacles for more nourishment to the poorly-organized and diseased tissue, thus presenting a greater surface of osmosis and capillary action?

Later in the article the doctor speaks of dehydration as lessening the sensitiveness of dentin. If the theory of sensitive dentin depended on the proper equilibrium being maintained between the external air and the pulp, this dehydration would cause traction on the nerves of the pulp and destroy the equilibrium which he maintains as necessary to painless dentin. It seems easier to explain this on the grounds of dehydration, or removal of chemical continuity by osmosis or capilarity, thus preventing a chemical from so quickly reaching the nerve end as it would if the tubuli were filled with a fluid.

As a summary of the preceding causes for the irritation of the nerve endings in the pulp, the theory already given (that sensation in teeth may be caused by either mechanical or chemical changes, setting up metabolism, which is the origin of all sensation in nerves), seems to satisfy all the phenomena connected with it.

The paper is a good one for discussion. And while I can not agree with it in many ways, it presents a field for work that should not be unproductive of good results.

Dr. H. A. Smith, Cincinnati: I was very much interested in the paper; it was a strong one, particularly from the standpoint of the writer.

There is just one thought that comes to me, and that is that the statement has been made and I may say somewhat dogmatically, that the dental tubuli are filled with a fluid.

I think the more recent investigations have proved it differently, and I might say that the stand taken by the writer is a very old doctrine, promulgated in the early days by Dr. Dickinson. More recent investigations in histology of the dentin show that the tubuli are filled with a protoplasmic mass, and I might say that this was long ago demonstrated by John Tomes, of England, and this with many other discoveries gave him everlasting fame, and for which he received a knighthood. Another point in reference to these tubuli filled with the fluid: analysis of dentin shows there is 10 percent of water only in the tooth substance, analysis also shows 25 percent of organic matter in the tubuli; if they were filled with fluid we would not have this large proportion of 25 percent organic and 75 percent of inorganic. I am only raising a doubt about the possibility of the existence of water.

The character of the contents of the tubuli has not been settled—that is whether it is a nerve tissue or not. Clinically they can hardly get away from the apprehension that it must be some form of nerve tissue or combination of same. If this is true of our clinical cases as well as microscopic investigation, the writer is at sea, especially on the theory given to-day.

A point in connection with the 25 percent organic matter. It is stated in the investigations of Seyler, Tomes and others that the proportion of water in the dentin itself is 10 percent; if we have 25 percent of organic matter and only 10 percent of water in the tissue, what is the rest of organic material—it must be solid tissue. Of course, there is organic matter in the walls of the dental tubes and in the radiating portions, but that is not enough to account for this difference. So that the contents of the tubuli would have to be somewhat solid; there would have to be a certain amount to bring up organic portion.

Dr. Custer: It was some ten or twelve years ago, possibly longer, that Dr. Andrews gave a name to those odontoblasts whose fibers extended in and filled the dental tubuli; he called them fibriloblasts. Now, so far as I have been able to observe, the fibriloblasts in distinction from odontoblasts are made up essentially of the same material as the odontoblasts, except that they have nerve function; it is also possible for simple protoplasm to be very highly-organized fibriloblasts. Their entire function, besides supplying nutrition, is one of conveying very high sensations, and it was that which was shown by Dr. Andrews that to my mind more clearly answers the question of sensitiveness of dentin. It is highly-organized protoplasm; it can not be simple water or fluid, but it is highly-organized protoplasm whose function is that simply of carrying nutrition, and with it the conveying of sensation.

Dr. Arnold: I should hesitate to decide the structure of the contents of the dental tubuli, and will simply make a point in reference to both questions as given by the essayist and question as propounded by Dr. Whinery. Both were quite ably presented, to my knowledge and estimation. In discussing the combination of the contents of dental tubuli we forget the benefits to be obtained in the treatment of sensitive dentin. From clinical experience we know too well that often, with the strongest dental anesthetic, the effect is very slow and tardy, in fact mostly so; now, again, we can use a milder therapeutic remedy and combine with it pressure and we get very active results, as shown by more recent introduction of a method known as "pressure anesthesia." It is a proper term, but, when we read of the method of the so-called pressure anesthesia as given by the man who introduced it, we learn nothing about pressure. The method was introduced by Dr. Morton, of New York, yet in his particular method he does not advocate pressure at all. There is, however, a method known properly as "pressure anesthesia;" you may combine some of the preparations, notably cocain, and apply with pressure and you do get most prompt obtundent conditions. We have a combination of mechanical and chemical agents working together. So that is strictly in clinical experience, whatever the contents may be we have little knowledge—not sufficient to prove the composition. Metabolic changes combined with mechanical dynamic force does give us a prompt action, the most satisfactory that I am aware of.

DR. SMITH: Since you have started me in this discussion I will continue; I want to defend myself. Now, in reference to vitality of enamel tissue. Has the enamel any vitality? Is it possible for it to have nutrition? If you recall the development of dentin, it is first and has a special organ. The enamel follows after, developed from its organ and has its special function.

When the enamel development is complete, the enamel organ disappears; the enamel as laid down on the dentin has no connection with the dentin, how then can the enamel receive nutrition?

It is so far from the pulp and has no connection with the pulp, so it is not possible for it to have sensation, and if it has no sensation, how can it have nutrition?

Now, these are problems for your consideration; I am only suggesting them.

We know that enamel is brittle when the dentin dies. How might this be explained?

Can we assume that there is a certain amount of circulation of fluids in the enamel?

Dr. Williams, of London, in his more recent investigations shows that the living matter in enamel is nothing that can be distinguished, but it is something which resembles organic tissue, has no structure and has no connection with the pulp except through abnormal dentin.

DR. W. T. MCLEAN: The theory advanced by the essayist is hard to disprove to my mind. The enamel does not get nutriment after development of teeth; it is just as Dr. Smith has stated. Anything useful for overcoming this pain should be worthy of our consideration. Operators should be more careful in the use of their instruments; use only the sharpest excavators and burs. Sharp instruments are what is required; do not use very dull burs and thereby be required to hold them on the tooth a long time to accomplish the purpose. I have seen them held on a tooth until the temperature must have been raised to at least 200°, and hence there is no doubt that it produces pain.

DR. M. H. FLETCHER: The position the essayist takes is certainly hard to disprove, but we may accept his theory that the dental tubes are filled with a fluid or a protoplasmic mass, from the fact that almost any substance in the way of metal even may be pressed into a different shape; it may be molded; take so soft a substance as protoplasm or fluid which fills a tube, it would not take very much pressure at one end of tube to produce its effect at the other. So I believe the essayist's position may be maintained on that particular topic.

As far as contents of the tubes is concerned I think Dr. Heise will bear me out on seeing some specimens last year in my office, where the contents of tubes show very plainly; in the center of the tube could be seen a fiber or material different from that on the side or at the periphery of the tube, showing out perfectly plain. Now, in support of what Dr. Smith says in regard to there being something more than fluid in the dental tubuli, I think this would go to support that theory. These sections that I speak of were dried specimens; after dehydrating they were cut with a file. If it were fluid which filled the tubes it would certainly have been driven out long before we filed or cut into the locality. The particular specimen was that of secondary growth of dentin; the tubuli ran in various directions, so my own experience with these cases would make me believe that the contents of the tube was of protoplasmic nature or gelatinous substance of some kind embedded in the center by the harder fiber, whether of nerve substances or not I am not able to say, and I do not know that any one has proven what it is. But the fact that the tube is filled with protoplasm does not disprove the position of the essayist, to my mind. Dr. Smith's position in reference to the enamel is well taken. As he said, the enamel organ disappears when the enamel is formed; it is built from the center out.

*Question:* Did I understand Dr. Smith to say that the enamel did not receive any nourishment?

Dr. Smith: I did not say so, dogmatically. It is built up in a manner entirely different from the dentin—from without inward—and it is a question I would not wish to decide. From a study of its formation and histology, it seems to me it can have no nutrition. How could it get it?

*Ques.:* If this is the case, how could our toe-nails and finger-nails grow if they received no nourishment?

Dr. Fletcher: Because there is a structure beneath them which gives them nourishment and they are pushed out as formed. It is different with enamel; it receives nourishment only while being formed from the enamel organ, then the organ disappears.

*Ques.:* At what age?

*Answer:* Before eruption.

*Ques.:* So, if a man lives for eighty years, you think the enamel will live all that time without receiving any nourishment?

*Ans.:* Yes, sir, that is possible.

*Ques.:* The point is this, I maintain that there can not be anything which will exist in the natural world unless it receives some nourishment.

*Voice:* How about dead teeth?

*Ans.:* They receive nourishment from the periosteum.

Dr. J. S. Cassidy: Not being a histologist I can not satisfactorily answer this question. A thought occurred to me about dehydrating the part and thus rendering it less able to conduct sensibility.

Dr. Junkerman tried to make the point that moisture present in the dentin hastened the ability to conduct sensation, whether a conductive material or not.

Whether the contents are liquid or semi-liquid seems to me to make practically no difference in the conductivity of the substance. If the contents of the tubuli is the only conducting material in the dentin, then the larger the tubuli the better the conducting power. But sensibility has existed in the tubuli when its canals have been nearly closed, so this refutes that. I may say I am not an authority on this, and I hate to be dogmatic. I believe any organized structure partakes somewhat of life as long as it exists or does its duty; enamel is an organized structure [But it is not vitalized, except by contact.—Ed.] as much so as any other part of the body, except that it does not have as much of organic matter. It is the product of vitality, as much so as any other highly-organized tissue of the body. I think the enamel does contain some life so long as peridental membrane exists, whether the pulp is devitalized or not, just in ratio of its necessity, however that may be. This is simply an idea of mine. There is no structure, the product of vitality, that does not retain a certain amount of vitality, or else it would soon be cast off.

Dr. Taft: I have no doubt that the enamel of all teeth with live pulps possesses a certain degree of vitality. All know that sometimes enamel in youth is comparatively soft, easily drilled into; it becomes harder as age progresses, and by and by it is very much more dense and resistant than in early life. Another point, all have seen that after the devitalization of the pulp the dentin and enamel also become more easy to break, and the rule is, in such cases as this, to cut down the borders of the enamel more extensively than we do in live teeth, because we have found we can put more reliance on the enamel borders of the live tooth and can depend more on the permanency of our work than a case where the pulp is dead. How often, after filling a lifeless tooth, do we find that after two, three or four years the enamel begins to crumble away, and it is finally the cause of recurrence of decay; how do we account for this, if there is no sustenance from the live tissues beneath it? The enamel organ is all gone, except so far as Nasmyth's membrane is concerned, it may be present. It is an exceedingly dense body and is for the protection of enamel early in life. As has been stated, the enamel is formed from within outward, and it remains in contact with the living structure of the dentin and receives its nutrient supply through the ends of the canals which perforate the dentin; the plasma contained in the canals passes out and comes into contact with the inner surface of the enamel and is taken up we say by inhibition, and supplies its wants. The points which the paper presented here were presented more than thirty years ago and the whole ground traversed over, but now and then this question is brought up again and has been discussed as it has been to-day without arriving at any definite conclusions. Dentin itself is susceptible of sensibility, independent, in the main, of the pulp. I infer that from this fact, oftentimes a layer or portion of the dentin under the decayed portion, after cutting away the decayed portion, is very sensitive, but pass through that and there is no longer any special sensitiveness. How is that? If the impression is made on the pulp, how is it that this condition of things exists?

My impression is that certain areas of dentin are susceptible when in an inflammatory condition; we call it inflammatory for accommodation or want of better expressiont—there is no better name for it. Any area is susceptible when in this condition, from the fact that it can be cut away as you know in many instances, and is absent entirely after cutting in toward the pulp; it is proof of my theory. I want nothing for the treatment of sensitive dentin except sharp excavators and burs.

Dr. Junkerman: Answering Dr. Taft's statement first. He brings up the question as to this soft layer of dentin referred to in the paper, which, to my mind, fully explains the reason why the dentin is sensitive; cut that layer away and there is no more sensitiveness, for the reason that the pressure produced upon this layer can be easily extended over the entire surface of the exposed dentin. A pressure produced on this soft tissue is extended over a great number of dental tubuli, the instant you take this away, by producing the same amount of force upon the layer underneath it, which is hard, without pain, in this case you do not strike as many of these little tubules. To my mind and in lieu of facts which have been proven by investigation, and until something better is offered, I must accept this theory. A theory does not mean that anything has been proven, but we all like to have some scheme upon which we can operate. If we do not know we like to theorize as to what we think the conditions are. To my mind this seems to satisfy all the principles better than any other theory that has been advanced. I may be entirely wrong, but until you give me a better one I must look at it in this light.

There is very little I can say, because nearly all the questions we have received have been answered by some other gentleman. Dr. Fletcher answered Dr. Smith's question as to contents of dental tubuli; I do not believe the paper asserts the fact that they are filled with water. It said it was fluid, whether water or semi-fluid, it matters not. If it is fluid the theory applies. In other words, protoplasm as Dr. Smith said, protoplasm is equivalent to water as far as theory is concerned.

The question was raised as to magnifying the force in the dental tubuli. The statement was made in the paper, "the longer the tubuli the greater the amount of force." I am not responsible for that statement, for that is one of the physical paradoxes, "the longer the tube the greater the pressure." If we place a tube in a barrel of water, and increase its length thirty or sixty times, the pressure will be magnified thirty or sixty times. That is what I mean by the magnification of force in the dental tubuli. I know that this theory is in some respects old, thirty years, as Dr. Taft says; he is perfectly correct about that. Since we dentists are of a mechanical nature the facts must be mechanical to be properly understood, so the object in promulgating this theory was to bring up a condition whereby we might bring it under the head of mechanical forces.

Dr. Taft: Dr. Junkerman assumes that that which I referred to was a layer of partially decomposed dentin; I distinctly stated that it was beneath this layer in such cases I referred to. It is beneath that partially decomposed dentin, after the latter has been cut away, that is so sensitive. Another instance in proof of the fact, take for instance the central incisors; we are all familiar with those spots of extreme sensitiveness, but if you have been a close observer, you know that with a bur or sharp excavator you can cut this away and all sensitiveness is gone.

